# *In Vitro* and *In Vivo* Effects of Suppressor of Cytokine Signalling 7 Knockdown in Breast Cancer: The Influence on Cellular Response to Hepatocyte Growth Factor

**DOI:** 10.1155/2014/648040

**Published:** 2014-08-04

**Authors:** Walid Sasi, Lin Ye, Wen G. Jiang, Anup K. Sharma, Kefah Mokbel

**Affiliations:** ^1^St. George's University of London, London SW17 0RE, UK; ^2^Cardiff University, Cardiff CF10 3XQ, UK; ^3^The London Breast Institute, The Princess Grace Hospital, London W1U 5NY, UK

## Abstract

*Purpose*. Suppressor of cytokine signaling 7 (SOCS7) is a member of the SOCS family and is known to interact with phospholipase C*γ*-1 (PLC*γ*-1), a key downstream mediator of the hepatocyte growth factor (HGF)/C-MET axis. Here, we report our observations of the effect of knocking down SOCS7 gene on the behaviour of breast cancer cells both *in vitro* and *in vivo* and to elucidate whether this involves HGF/C-MET pathway using the PLC*γ*-1 blocker U73122. *Methods*. MCF7 and MDA-MB-231 breast cancer cells were transfected with anti-SOCS7 ribozymal transgene, to create sublines with SOCS7 knockdown. The *in vitro* growth and migration of the cells were evaluated in basic conditions and with HGF and U73122 treatment using growth assays, scratch-wound, and electrical cell impedance sensing (ECIS) migration assays. MCF7 and MDA-MB-231 *in vivo* tumour xenograft growth were also studied. *Results*. Basal *in vitro* growth and migration of both cellular lines and the *in vivo* MCF7 xenograft growth were significantly enhanced with SOCS7 knockdown. *In vitro* HGF treatment has further influenced the growth and migration when SOCS7 gene was knocked-down in both cellular lines (*P* < 0.05). PLC*γ*-1 pharmacological inhibition of the HGF/C-MET cascade during their *in vitro* growth and migration seemed to only occur when SOCS7 gene was knocked down. *Conclusions*. We report a unique regulatory role for SOCS7 in controlling the malignant behaviour of breast cancer lines MCF7 and MDA-MB-231 *in vitro* and the MCF7 tumour xenografts *in vivo*. We also report a regulatory role for SOCS7 during the *in vitro* HGF-induced growth and migration in these cells as HGF treatment and SOCS7 loss have synergistically enhanced these functions. This SOCS7 knockdown-attributed effect could be due to a precise anti-PLC*γ*-1 role.

## 1. Introduction

Suppressor of cytokine signalling 7 (SOCS7) is a member of the SOCS family which has been implicated in the regulation of many intracellular molecular mechanisms downstream of cytokine and growth factor receptors. Among these regulatory functions of the SOCS family is a variably characterised tumour suppressing role of some of its members. Only recently a tumour suppressing role was described for SOCS7 particularly in breast cancer [1], and this intriguing role is still under investigation.

Hepatocyte growth factor (HGF) is a multifunctional cytokine that elicits diverse responses in different cells and tissues. Much evidence now points to its drive of carcinogenesis and cancer invasion and metastasis. HGF and its receptor, C-MET, are both critical mediators of breast cancer progression, and, like in many other solid organ cancers, both HGF and C-MET are highly expressed in breast cancer [2–5]. HGF and C-MET expression correlates with mammary tumour pathology, showing lowest expression levels in normal tissue and benign hyperplasia while increasing in ductal carcinoma* in situ* and showing highest expression in invasive breast carcinomas [6]. High HGF and C-MET expression levels are now considered as independent prognostic indicators for poor patient survival [4, 7].

In addition to its predictive expression in human mammary tumours, HGF is a potent mammary tumour inducer in mice, as targeted expression of HGF in mouse mammary epithelium was found to lead to metastatic adenocarcinomas [8]. Further reports showed that HGF/C-MET downstream intermediate phospholipase C*γ*-1 (PLC*γ*-1) overexpression was also observed in breast cancer [9], and specific PLC*γ*-1 inhibition was found to block breast cancer invasiveness [10].

Together these data support a role for HGF/C-MET/PLC*γ*-1 route as a direct mediator in breast cancer progression, thus making it a good target for therapeutic intervention.

Historically, the SOCS7 variant (NAP4) was found to directly interact with PLC*γ*-1, and other intermediates such as NcK and Ash through the SOCS7-SH2 domain [11].

NcK is a cytoplasmic receptor tyrosine kinase adaptor molecule [12, 13] which is involved in HGF/C-MET/PLC*γ* signalling [14, 15].

Further reports have illustrated these SOCS7-NcK interactions [16, 17] and a possible involvement of SOCS7 in cell cycle arrest and in initiating the p53 apoptotic pathway [16].

The mediator Grb2 (otherwise known as Ash) is also activated during the HGF/C-MET signalling leading to the activation of downstream MAPK pathway involved in the cellular proliferation and differentiation [18, 19] and can also be involved in cellular invasion and motility through activation of downstream FAK pathway [19]. SOCS7 can interact with Grb2 at this level [11, 17, 20].

SOCS7 can also directly interact with p85, the regulatory subunit of the HGF/C-MET downstream PI3K-AKT cascade [16, 21], and JAK-STAT regulation by SOCS7 is also possible, although not specifically reported in HGF/C-MET signalling. For instance, as both STAT3 and STAT5 can be activated downstream of HGF/C-MET through GAB1 [19], SOCS7 can inhibit JAK2-STAT3 [11, 17, 20, 22], is known to interact with STAT5* in vitro* [23], and can alter the nuclear localisation of pSTAT5 [24, 25].

Taken together, the above reports suggest a possible multiregulatory involvement of SOCS7 in HGF/C-MET signalling. We here aimed to observe the effect of SOCS7 knockdown on the behaviour of breast cancer both* in vitro* and* in vivo* and to investigate whether SOCS7 knockdown in breast cancer cells MCF7 (ER +ve) and MDA-MB-231 (ER −ve) can affect their* in vitro* growth and migrational responses when treated with HGF.

We hypothesised that SOCS7 is a negative regulator of HGF effects, therefore predicting an additive effect of HGF treatment and SOCS7 knockdown. A series of functional assays were conducted in which we utilised HGF and the specific pharmacological blockade of PLC*γ*-1 by using the inhibitor U73122, to investigate whether SOCS7 regulates the HGF/C-MET/PLC*γ*-1 axis by interacting with PLC*γ*-1 as U73122 blockade of PLC*γ*-1 would mitigate the anti-PLC role of SOCS7. However, we did not intend to look into the molecular details of SOCS7 interactions within this axis.

## 2. Materials and Methods

### 2.1. Cell Lines

Human breast cancer cells MCF7 and MDA-MB-231 were purchased from the American Type Culture Collection (ATCC, Maryland, US) and maintained in Dulbecco's modified Eagle's medium supplemented with 10% foetal calf serum (PAA Laboratories Ltd., Somerset, UK), streptomycin and penicillin. The cells were incubated at 37°C, 5% CO_2_ and 95% humidity.

### 2.2. HGF and U73122

HGF was purchased from Sigma-Aldrich (Poole, UK) and used in concentration of 40 ng/mL. PLC*γ*-1 inhibitor (U73122) was purchased from Calbiochem (Merck Chemicals Ltd., Nottingham, UK) and used in concentration of 200 nM/L. A single dose of both agents (HGF and U73122) was used to observe for occurrence of the effect(s) on the cellular* in vitro* functions rather than to quantify the magnitude of any possible effect. Targeting the PLC*γ*-1 isoform with siRNA is probably more specific than U73122, as the later has more pan anti-PLC function including other PLC isoforms. However, in the context of this study, we sought to observe the HGF stimulus effect through the HGF/C-MET/PLC*γ*-1 cascade. Therefore, U73122 was considered a reliable blocker of the PLC*γ*-1 activity, as other PLC isoforms are not known to be involved in this pathway.

### 2.3. RNA Preparation and RT-PCR

Two groups of primers were designed using Beacon Designer (PREMIER Biosoft International, Palo Alto, CA, USA). The first group was designed to amplify the coding sequence of SOCS7 gene (Table 1); and the second category was designed according to the secondary structure of SOCS7 gene transcript and was used to synthesize a hammerhead ribozyme for the gene silencing study (Table 2). Total RNA was extracted from cells using RNA extraction kit (AbGene Ltd., Surrey, England, UK) and its concentration quantified using a spectrophotometer (Wolf Laboratories, York, England, UK). cDNA was synthesized using a first strand synthesis with an oligodt primer (AbGene, Surrey, UK). The polymerase chain reaction (PCR) was performed with the following conditions: 5 min at 95°C and then 20 s at 94°C-25 s at 56°C, 50 sat 72°C for 36 cycles, and finally 72°C for 7 min. GAPDH was amplified and used as a house-keeping control. PCR products were then separated on 0.8% agarose gel, visualized under UV light, photographed using Unisave camera (Wolf Laboratories, York, England, UK) and documented with Photoshop software.

### 2.4. Construction of Hammerhead Ribozyme Transgenes Targeting Human SOCS7 (*hSOCS7*)

To knockdown* hSOCS7* gene (GenBank Accession: NM_014598.2), by using a hammerhead ribozyme transgene, we designed primers according to secondary structure of the gene generated by using Zuker's RNA mFold programme, targeting at a specific GUC or AUC site (Table 2). We synthesised ribozymes with a Touchdown PCR procedure and cloned ribozymes into a mammalian expression pEF6/V5-His-TOPO plasmid vector (Invitrogen Ltd., Paisley, UK). SOCS7 ribozyme transgenes 1, 2, and 3 and control plasmid vectors were then transfected into studied cells, respectively, using electroporation. For electroporation, we utilized the Easy Jet Plus system (Flowgen, Staffordshire, UK), which passed a voltage of up to 310 volts across the cells to produce small perforations in the cell wall integrity, thus allowing passage of plasmid DNA across cell membranes to be integrated into the cells.

For a transfection, 3 pg of plasmid DNA was added to resuspended (−1 × 10~) cells and mixed. The mixture was left to stand at room temperature for 2 to 5 minutes. The mixture was then transferred into an electroporation cuvette (Euro Gentech, Southampton, UK) ready for electroporation. The cuvette was loaded into the electroporator and a pulse of electricity (250–310 volts, depending on cell types) was passed through the cuvette. The mixture was then immediately (within 10 seconds) transferred into 10 mL of prewarmed culture medium (must be within 30 seconds). This reaction was then cultured under the usual incubation conditions.

**Figure 1:**
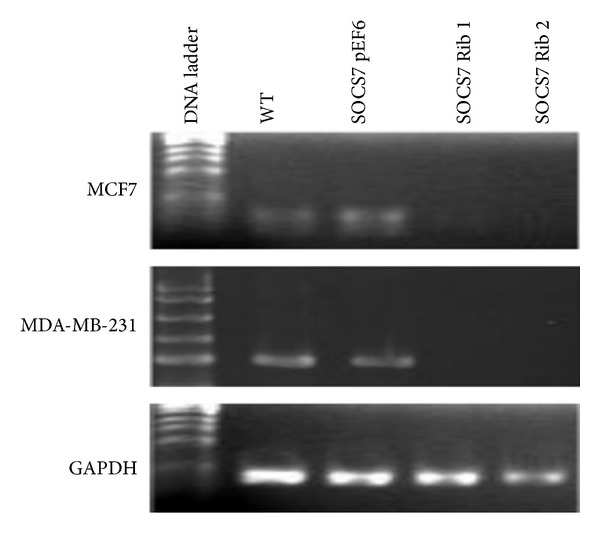
RT-PCR. Knocking down SOCS7 in MCF7 and MDA-MB-231. The top two panels show the absence of SOCS7 cDNA bands in cells transfected with SOCS7 ribozymes 1 and 2 vectors. GAPDH was used as an internal control in the bottom panel.

After 3-4 weeks selection with the antibiotic blasticidin (5 *μ*g/mL), a stable cell line with the transgene was verified by using RT-PCR for the success of knockdown before being used in our experiments (Figure 1). This method has been extensively used and reported previously in our laboratory [22].

Six cellular sublines were established: SOCS7 knockdown cells (MCF7^ΔSOCS7^ and MDA-MB-231^ΔSOCS7^), plasmid-only control cells (MCF7^pEF6^ and MDA-MB-231^pEF6^), and the wild-type, MCF7^WT^ and MDA-MB-231^WT^. The knockdown and plasmid-control cells were always kept in a maintenance medium which contained 0.5 *μ*g/mL blasticidin. Scrambled ribozyme controls were used to discount any changes to the gene expression profile that might result from the ribozymal delivery method. We compared cDNA bands from cells transfected with scrambled ribozyme control and untransfected cells and found no changes caused by ribozyme delivery.

### 2.5. *In Vitro* Cell Growth Assay

Cells suspension was added into 96-well plates [2500 cells in 100 *μ*L HEPES (4-(2-hydroxyethyl)-1-piperazineethanesulfonic acid) buffering medium per well]. For control wells, additional 100 *μ*L of HEPES medium was added. Cells were allowed to adhere to plate surface prior to treatment. 100 *μ*L of an HGF containing HEPES solution (40 ng/mL) was added to the HGF treatment wells. When the PLC*γ*-1 inhibitor U732122 (1 *μ*M) was used, cells were treated for 15 minutes prior to the addition of HGF (40 ng/mL). These plates were incubated for 2 hours at 37°C before making the initial measurement (time point 0). Cells were fixed in 10% formaldehyde on the day of plating and daily for the subsequent 4 days. 0.5% crystal violet (w/v) was used to stain cells. Following washing with dH_2_O twice and drying, the stained crystal violet was dissolved with 10% (v/v) acetic acid and the absorbance of the dissolved dye, corresponding to the number of viable cells, was determined at a wavelength of 540 nm using an ELx800 spectrophotometer (BIO-TEK, ELx800, Wolf Laboratories, York, England). Normalised cellular growth (proliferation) rate was determined by the equation: (Absorbance at Day 4/Absorbance at Day 0) × 100%, where Day 0 is the day of cell plating.

### 2.6. *In Vitro* Migration Scratch—Wounding Assay

Cells at a density of 35,000 cells/200 *μ*L/well were seeded into 24-well plates and allowed to reach near confluence by incubation at 37°C for 24 hours, then scratched with a pipette tip to create wound size of approximately 200 *μ*m, and washed twice in PBS to remove floating cells. When the PLC*γ*-1 inhibitor U732122 (1 *μ*M) was used, cells were treated for 15 minutes prior to the addition of HGF (40 ng/mL). The cells were photographed at intervals using an inverted microscope; the sizes of the wounds were subsequently analysed with the TScratch software (ETH Zurich, 2008).

### 2.7. Electric Cell Substrate Impedance Sensing (ECIS) Assay

ECIS-1600R model (Applied Biophysics, Inc., Troy, NY) was used for migration modeling in wounding analysis [23]. 8W10E arrays were used. Each of the 8 wells contains ten circular 250 *μ*m diameter active electrodes connected in parallel on a common gold pad. Each well has a substrate area of 0.8 cm^2^ and a maximum volume of 600 *μ*L. On average, with a confluent cell layer, approximately 500–1000 cells will be measured by the electrodes.

Following treating the array surface with a cysteine solution (10 mM), the arrays were incubated in a serum-free medium (± HGF ± U73122) for 1 hour. The same number of the respective cells (250,000 per well) was added to each well. When confluence was reached, the monolayer was electrically wounded at 6 V AC and 4000 KHz for 30 seconds.* In vitro* migration rate was determined using the method previously described [23].

### 2.8. *In Vivo* Growth Assay (for Assessment of* In Vivo* Development of Mammary Tumours)

Athymic nude mice (Nude CD-1) of 4–6 weeks old were purchased from Charles River Laboratories, Kent, UK, and maintained in filter-toped units. Animals planned for MCF7 cell inoculation were implanted with oestrogen pellets made of a mixture of 2 mg of E2 and 18 mg of cholesterol.

Breast cancer cells in culture flasks were first washed using sterile BSS and treated using EDTA-Trypsin buffer. After removing EDTA-Trypsin and washing, the single cell suspension was prepared using serum free medium which also contained 0.5 mg/mL Matrigel. The cell number in the suspension is 5 × 10^6^/mL. 100 *μ*L of this cell suspension (containing 0.5 million cells) was injected subcutaneously at the left scapula area as previously described at our laboratory [26].

Four groups were included: MCF7 empty plasmid vector/control transfection (pEF6), MCF7 with SOCS7 gene knockdown (ΔSOCS7), MDA-MB-231 control transfection (pEF6), and MDA-MB-231 with SOCS7 gene knockdown (ΔSOCS7). Each tumour group included 6 athymic nude mice. Mice were weighed and tumour sizes measured twice weekly for 4 weeks. Mice with weight loss over 25% or tumour size larger than 1 cm in any dimension were terminated according to the UK Home Office and UKCCCR guidelines.

The volume of the xenograft tumour was determined using the formula:

(1) $$\begin{array}{c} \text{Tumour}\;\;\text{Volume}=0.523\times {\text{Width}}^{2}\times \text{Length}. \end{array}$$

At the conclusion of the experiment, animals were terminally anaesthetised; primary tumours were dissected, weighed and frozen at −80°C. Parts of the primary tumours were fixed for future histological examination. Factors such as tumour tissue oedema, necrosis, and the amount of connective tissue will be further studied following a detailed histological examination. This is in order to verify and confirm that the tumour volume results correlate with the lean tumour mass.

### 2.9. Statistical Analysis

Statistical analysis was performed using SPSS version 16. Normality of data was tested by K-S and Shapiro-Wilk tests, and homogeneity of variances was tested by Levene's test. For normal data, ANOVA and* post-hoc* analysis was used for multiple comparisons, and two-tailed student *t*-test for single two-sample comparisons. For nonnormally distributed data, Kruksall-Wallis analysis was used for multiple comparisons, and Mann-Whitney* U* test was used for single two-sample comparisons. Results of cell growth and ECIS assays were presented as mean ± SE of three independent experiments. RT-PCR and ECIS figures were taken from representative experiments.

## 3. Results

### 3.1. Verification of SOCS7 Gene Knockdown (Figure 1)

Due to the low expression of SOCS7 protein and the lack of a suitable antibody, we were unable to detect SOCS7 by immunoblot. However, SOCS7 gene knockdown in both MCF7 and MDA-MB-231 cells was confirmed at mRNA level using RT-PCR. Successful knockdown was achieved using the designed anti-SOCS7 ribozymes 1 and 2.

### 3.2. *In Vitro* Growth Assays (Tables 3 and 4; Figure 2)

At basic conditions and with SOCS7 knockdown, the MCF7 and MDA-MB-231 cellular* in vitro* growth appeared to be significantly more than the control and wild-type growth.

In addition, HGF has produced a significant influence on all MCF7 sublines. For instance, after 4 days of incubation,* in vitro* growth of HGF-stimulated MCF7^WT^ cells was significantly larger than that of the unstimulated similar subline or MCF7^pEF6^ (control) cells incubated for the same duration. This was also true with HGF-stimulated MCF7^pEF6^ cells compared to unstimulated similar and wild-type cells. Additionally, MCF7^ΔSOCS7^ cells treated with HGF have shown more growth than similar untreated cells.

MCF7^ΔSOCS7^ cellular growth following HGF treatment was larger than that of HGF-treated wild-type and control cells. Pretreatment with the PLC*γ*-1 inhibitor (U73122) appeared to abolish this growth difference as MCF7^ΔSOCS7^ cellular growth was no longer significantly larger than that of MCF7^WT^ and MCF7^pEF6^ cells when all three sublines were treated with HGF and U73122.

Treatment of cells with HGF and U73122 has resulted in abrogation of these HGF-induced growth effects in each subline, and in the case of MCF7^ΔSOCS7^, U73122 seemed to significantly block any HGF effect on their growth [1.3(0.25) with HGF versus 0.85(0.02) with HGF and U73122; *P* = 0.002].

In the case of MDA-MB-231 cells, HGF has produced a significant influence on all their sublines. For instance, after 4 days of incubation,* in vitro* growth of HGF-stimulated MDA-MB-231^WT^ cells was significantly larger than that of a untreated similar subline or MDA-MB-231^pEF6^ (control) cells incubated for the same duration. This was also true with HGF-stimulated MDA-MB-231^pEF6^ cells compared to unstimulated similar and wild-type cells. Additionally, HGF-treated MDA-MB-231^ΔSOCS7^ cells have shown more growth than similar untreated cells.

MDA-MB-231^ΔSOCS7^ cellular growth with HGF was larger than the stimulated wild-type and control cells. As with MCF7 cells, adding U73122 has abrogated this HGF-mediated growth induction as MDA-MB-231^ΔSOCS7^ cellular growth was no longer significantly larger than that of MDA-MB-231^WT^ and MDA-MB-231^pEF6^ cells.

### 3.3. *In Vitro* Scratch-Wound Migration Assays (Tables 5 and 6, Figures 3 and 4)

At basic conditions and with SOCS7 knockdown, the MCF7 and MDA-MB-231 cellular* in vitro* migration appeared to be significantly more than the control and wild-type migration. With HGF treatment, stimulated MCF7^ΔSOCS7^ migration was significantly better than that of stimulated MCF7^pEF6^ cells. A positive synergistic influence of HGF on each subline was also noted. For instance, HGF-treated MCF7^ΔSOCS7^ migration was better than that of the similar unstimulated subline, and HGF-treated MCF7^pEF6^ migration was significantly better than that of the similar unstimulated subline. The pretreatment with U73122 has significantly blocked the stimulatory effect of HGF on MCF7^ΔSOCS7^ migration but not that of the MCF7^pEF6^ cells [59.8(20.3) versus 31.9(19.6); *P* = 0.005 and 55.9(17.2) versus 54.7(15.5); *P* = 0.9,  resp.].

With HGF treatment, stimulated MDA-MB-231^ΔSOCS7^ migration was significantly better than that of stimulated MDA-MB-231^pEF6^ cells. As with MCF7 cells, a positive synergistic influence of HGF on each subline was again noted. For instance, stimulated MDA-MB-231^ΔSOCS7^ migration was better than that of the similar untreated subline and stimulated MDA-MB-231^pEF6^ migration was significantly better than that of the similar untreated subline. The pretreatment with U73122 has significantly abrogated the stimulatory effect of HGF on MDA-MB-231^ΔSOCS7^ migration but not that of the MDA-MB-231^pEF6^ cells [61.2(16.2) versus 34.87(3.8); *P* = 0.007 and 71.8(4) versus 58.5(2); *P* = 0.87,  resp.].

### 3.4. *In Vitro* Electrical Cell Impedance Sensing (ECIS) Assays (Tables 7 and 8, Figures 5 and 6)

Unstimulated MCF7^ΔSOCS7^ migration was significantly more than that of both unstimulated MCF7^WT^ and unstimulated MCF7^pEF6^ cells. HGF-treated MCF7^ΔSOCS7^ migration was significantly more than that of treated control cells. Although the overall HGF effect on MCF7 cellular migration was stimulatory, the HGF effect was only significant enough in MCF7^pEF6^ migration but not in MCF7^ΔSOCS7^. HGF-treated MCF7^pEF6^ migration was significantly more than that of the similar untreated subline [62.9(19.3) versus 37(17.6); *P* = 0.026], while HGF-treated MCF7^ΔSOCS7^ migration was slightly but insignificantly more than that of similar untreated cells. The addition of U73122 has significantly limited the small stimulatory effect of HGF on MCF7^ΔSOCS7^ migration but not that of the MCF7^pEF6^.

Similar to MCF7 cells, untreated (basal) MDA-MB-231^ΔSOCS7^ migration was significantly more than that of both untreated wild-type and control cells.

HGF-treated MDA-MB-231^ΔSOCS7^ migration was significantly more than that of treated MDA-MB-231^pEF6^ cells but there was also a positive influence of HGF on each subline. For instance, stimulated MDA-MB-231^ΔSOCS7^ migration was more than that of the similar un-stimulated cells and stimulated MDA-MB-231^pEF6^ migration was significantly more than that of the similar unstimulated cells. The addition of U73122 has significantly abrogated the stimulatory effect of HGF on the migration of both sublines of MDA-MB-231. For instance, the migration of MDA-MB-231^pEF6^ was significantly limited with HGF and U73122 pre-treatment compared to similar cells treated with HGF only [54.2(10.2) versus 104.2(36.1); *P* = 0.046], and the migration of MDA-MB-231^ΔSOCS7^ was also significantly limited with HGF and U73122 pretreatment compared to similar cells treated with HGF only [59.3(58.8) versus 222(48.1); *P* < 0.001].

### 3.5. *In Vivo* Growth of the Mammary Tumours (Tables 9 and 10, Figure 7)

In the CD-1 athymic nude mice model, it was shown that MCF7^ΔSOCS7^ tumour group grew at a significantly faster pace compared to the control (MCF7^pEF6^) tumours. The difference of tumour size was seen from early time points (from 7 days onwards), and the overall difference between MCF7^ΔSOCS7^ tumours and transfection control tumours were highly significant (*P* < 0.001 by two-way ANOVA).

In regards to MDA-MB-231 mammary tumours, there was no statistically significant overall difference in the growth of MDA-MB-231 tumours with SOCS7 knockdown compared to that of the transfection control group, even though it appears that the control group tumours have grown marginally bigger (*P* = 0.057 by two-way ANOVA).

## 4. Discussion

SOCS7, like other SOCS family members, is known to be expressed by MCF7 and MDA-MB-231 cells [27, 28]. Under basic conditions, several* in vitro* functions of MCF7 cells seem to be significantly affected with SOCS7 knockdown, namely, their growth and migration. Both* in vitro* growth and migration of MCF7 cell lines were enhanced with SOCS7 knockdown, as was their* in vivo* xenograft growth in the mouse model. This strongly suggests a critical role for SOCS7 in regulating these functions in MCF7 cells.

Data are less clear from MDA-MB-231 experiments. Although their* in vitro* growth was enhanced with the SOCS7 knockdown, this was not mirrored during* in vivo* growth studies. This is a reminder that* in vitro* conditions may not represent the true pathophysiological environment in the host body of the mouse model with all its growth factor and cytokine crosstalk which may have led to a functional redundancy of SOCS7 in these cells. Their* in vitro* migration, however, was enhanced by SOCS7 knockdown under basal conditions.

Recent studies suggested an influential role of SOCS7 in regulating cellular division through its involvement in the Septin-SOCS7-NcK axis. Cytoplasmic SOCS7 was found to be involved in binding and translocating the adaptor protein NcK to the nucleus to inhibit cellular division initiating cell cycle arrest, in response to conditions such as DNA damage [16]. NcK nuclear accumulation in turn leads to the activation of p53 and its linked pathways. Hence, the loss of SOCS7 can also be linked—through loss of p53 cascade activation—to increased cancer cell proliferation. This uncontrolled* in vitro* MCF7 and MDA-MB-231 growth with SOCS7 knockdown might therefore be the result of an unchecked cellular division owing to the loss of SOCS7-NcK and p53 regulatory role in cellular division.

In this study we hypothesised a regulatory role for SOCS7 in HGF/C-MET signalling in breast cancer based on its multiple interactions with intermediate molecules downstream of the C-MET receptor. The most important of these is the PLC*γ*-1 and NcK, both were reported to form a complex with the activated receptor [14], and our previous data showed a specific anti-PLC*γ* role for SOCS7 in IGF-I signalling [28]. The knockdown of SOCS7 would not have increased the C-MET or the PLC*γ*-1 expression as their expression in the wild-type MCF7 and MDA-MB-231 cells is already strong [29, 30].

Our observations here do indeed support this hypothesis in the* in vitro* environment. For instance, we observed that HGF has produced a positive influence on the growth of all MCF7 sublines (control and knockdown) but produced more significant influence on the MCF7^ΔSOCS7^ cells compared to HGF-treated wild-type or control cells. These growth effects appeared to be affected by pretreatment with the PLC*γ*-1 inhibitor, U73122, as any significant HGF-induced growth difference between MCF7^ΔSOCS7^, control and wild-type cells has become negligible. Similar observations were seen during MDA-MB-231* in vitro* growth assay. These observations may indicate that in addition to the expected HGF positive effect on the growth and proliferation of MCF7 and MDA-MB-231 cells* in vitro*, this effect was synergistically enhanced with the knockdown of SOCS7 gene, but as this growth difference between knockdown sublines and the control sublines became abrogated with U73122, this may indicate a specific role for SOCS7 in the HGF/PLC*γ* proliferation axis.

Using scratch-wound and ECIS assays, we observed that MCF7^ΔSOCS7^ and MDA-MB-231^ΔSOCS7^ cells under the HGF stimulus had demonstrated a more enhanced migration than did stimulated control cells (*P* < 0.05). We also observed a rate of migration of MCF7^ΔSOCS7^ cells slightly higher (but with no statistical significance) following HGF treatment than that of similar unstimulated cells and significantly higher than that of similar cells treated with HGF and U73122. MDA-MB-231^ΔSOCS7^ migration followed a very similar pattern with all differences statistically significant (*P* < 0.05). These migration data showed an additive influence of HGF treatment and SOCS7 knockdown on the* in vitro* migration of both breast cancer lines. They also showed that such additive influence due to SOCS7 knockdown was lost with U73122 treatment, which may point to a precise anti-PLC*γ*-1 role for SOCS7.

## 5. Conclusion

SOCS7 knockdown can result in increased MCF7 and MDA-MB-231 basal cellular growth and migration* in vitro* and can positively influence the growth of MCF7* in vivo* tumour xenografts in nude athymic mice. This is suggestive of a tumour suppressive role for this molecule in MCF7 breast cancer cells. No similar growth results were shown from the MDA-MB-231 cellular* in vivo* growth observations. SOCS7 knockdown, however, has enhanced the MDA-MB-231 cellular migration.

We also postulate a significant involvement of SOCS7 in the HGF/PLC*γ*-1 regulation. SOCS7 loss has resulted in the amplification of HGF/C-MET growth and migrational signalling in the two studied breast cancer cell lines, but pharmacological blockade of PLC*γ*-1 enzymatic activity has mitigated this amplified signalling. This could mean that SOCS7 is involved very precisely in the regulation of PLC*γ*-1 function.

## Figures and Tables

**Figure 2:**
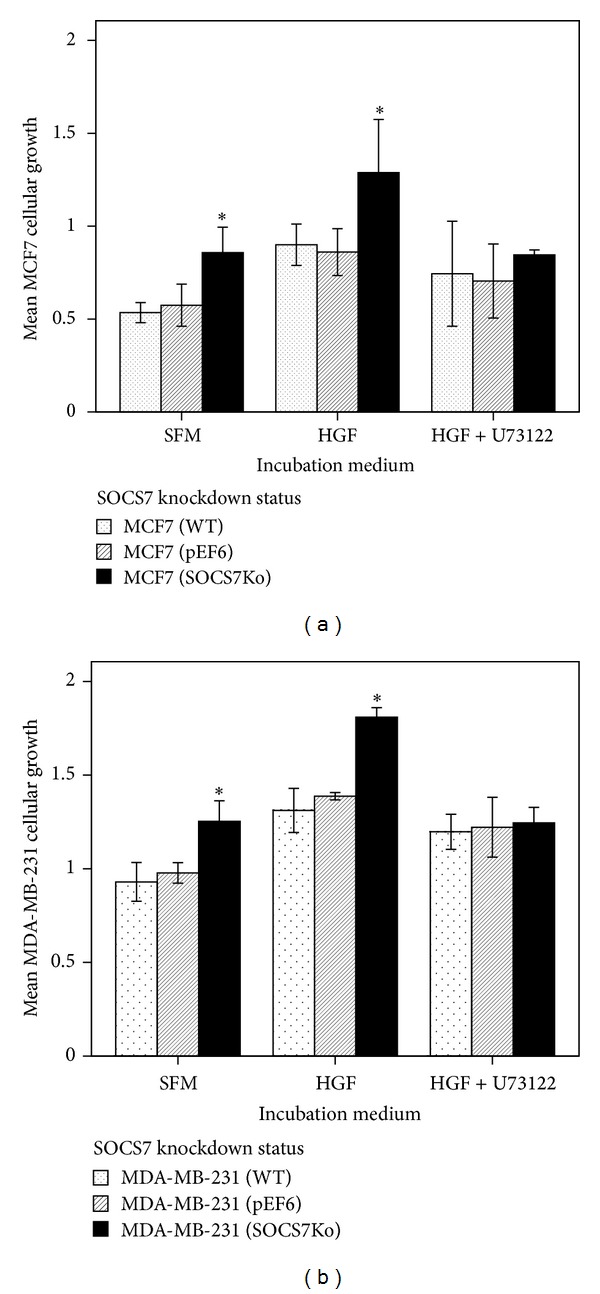
(a) MCF7* in vitro* growth (96 hours). (b) MDA-MB-231* in vitro* growth (96 hours).

**Figure 3:**
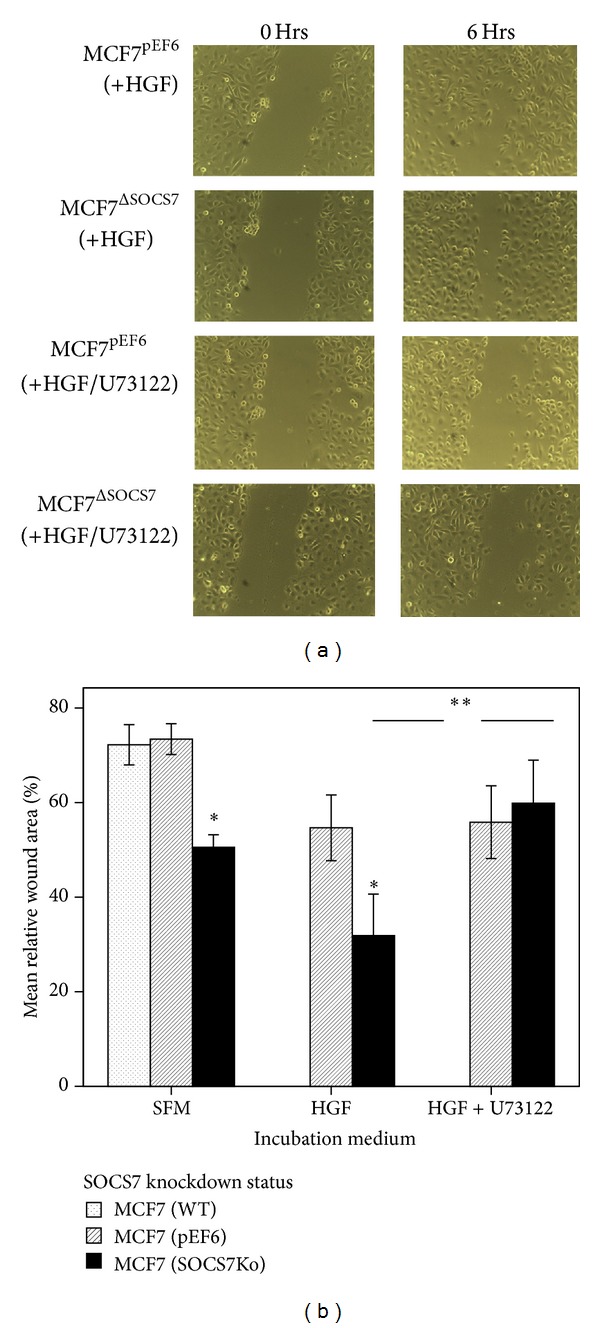
(a) MCF7 scratch wound assay—with HGF/U73122. (b) Relative scratch-wound width area (%) of the MCF7 monolayer.

**Figure 4:**
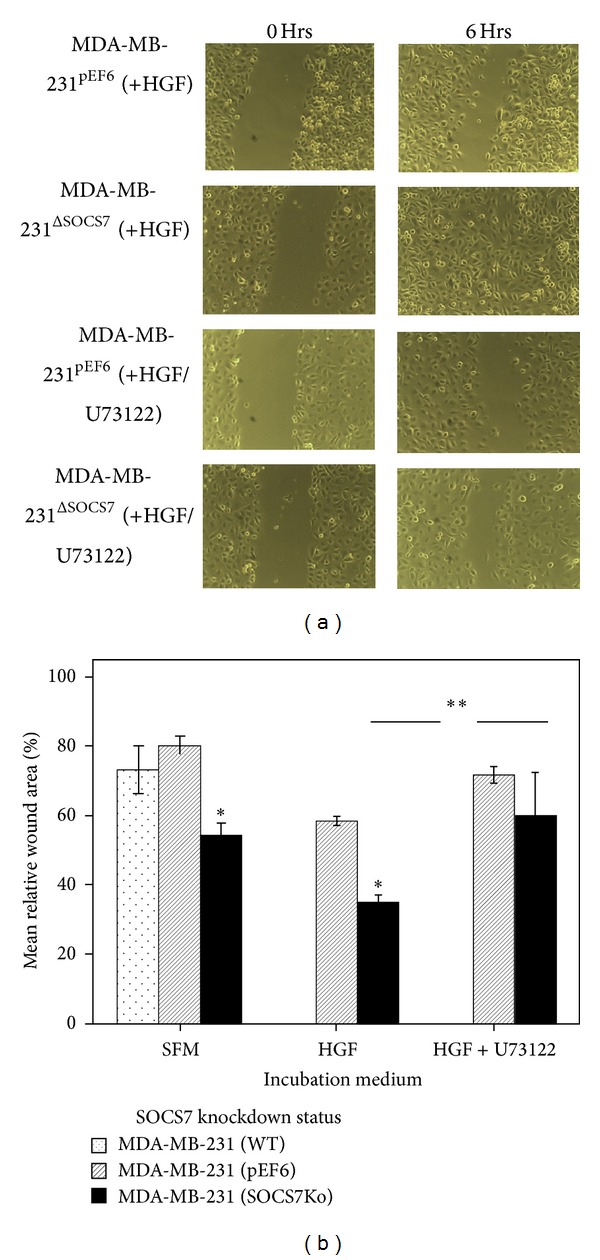
(a) MDA-MB-231 scratch wounding assay—with HGF/U73122. (b) Relative scratch-wound width area (%) of the MDA-MB-231 monolayer.

**Figure 5:**
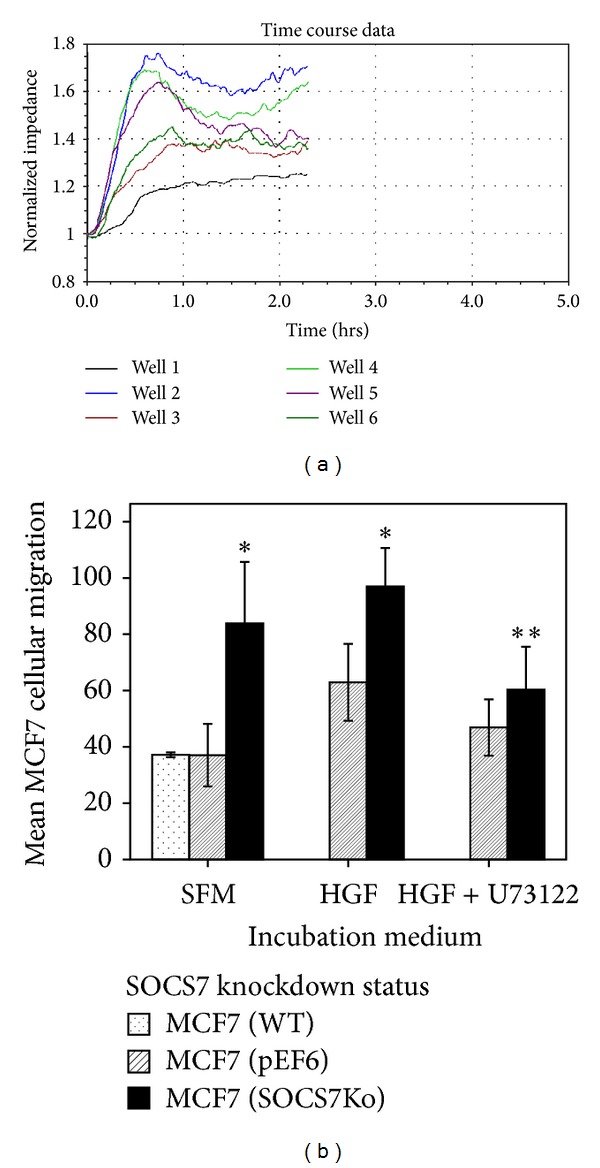
(a) A representative ECIS wounding experiment to study* in vitro* migration rate of MCF7. ECIS time course following electrical wounding (6 V for 30 Sec). Wells: (1) MCF7^WT^ (untreated); (2) MCF7^ΔSOCS7^ (+HGF); (3) MCF7^pEF6^ (+HGF/U73122); (4) MCF7^ΔSOCS7^ (untreated); (5) MCF7^pEF6^ (+HGF); and (6) MCF7^ΔSOCS7^ (+HGF/U73122). (b)* In vitro* migration of MCF7 sublines with and without HGF.

**Figure 6:**
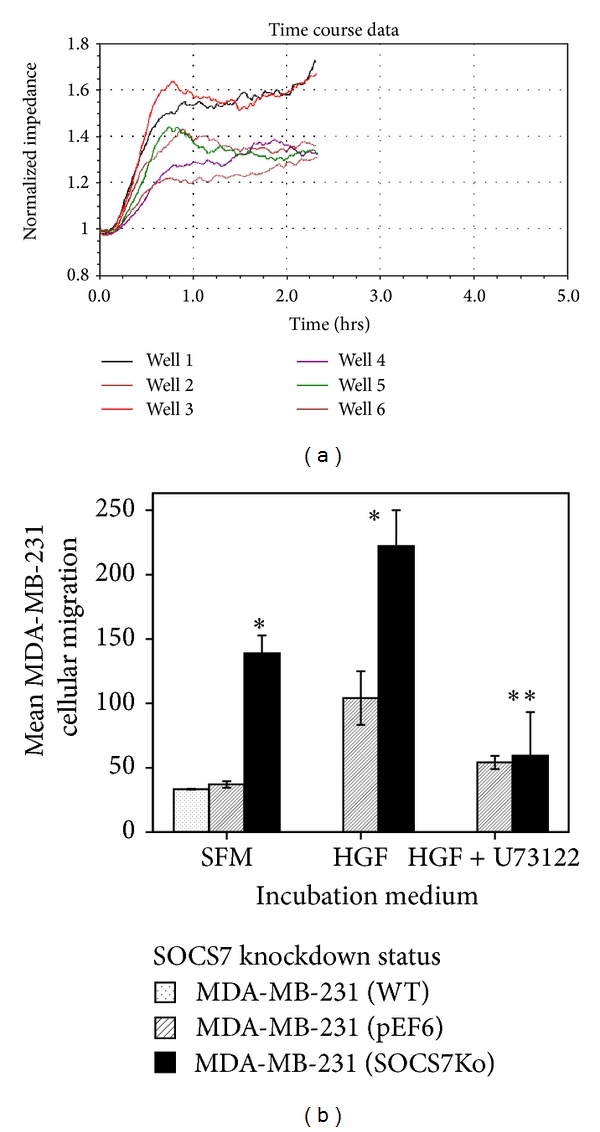
(a) A representative ECIS wounding experiment to study* in vitro* migration rate of MDA-MB-231. ECIS time course following electrical wounding (6V for 30 Sec). Wells: (1) MDA-MB-231^ΔSOCS^ (untreated); (2) MDA-MB-231^ΔSOCS7^ (+HGF/U73122); (3) MDA-MB-231^ΔSOCS7^ (+HGF); (4) MDA-MB-231^pEF6^ (+HGF/U73122); (5) MDA-MB-231^pEF6^ (+HGF); and (6) MDA-MB-231^WT^ (untreated). (b)* In vitro* migration of MDA-MB-231 sublines with and without HGF.

**Figure 7:**
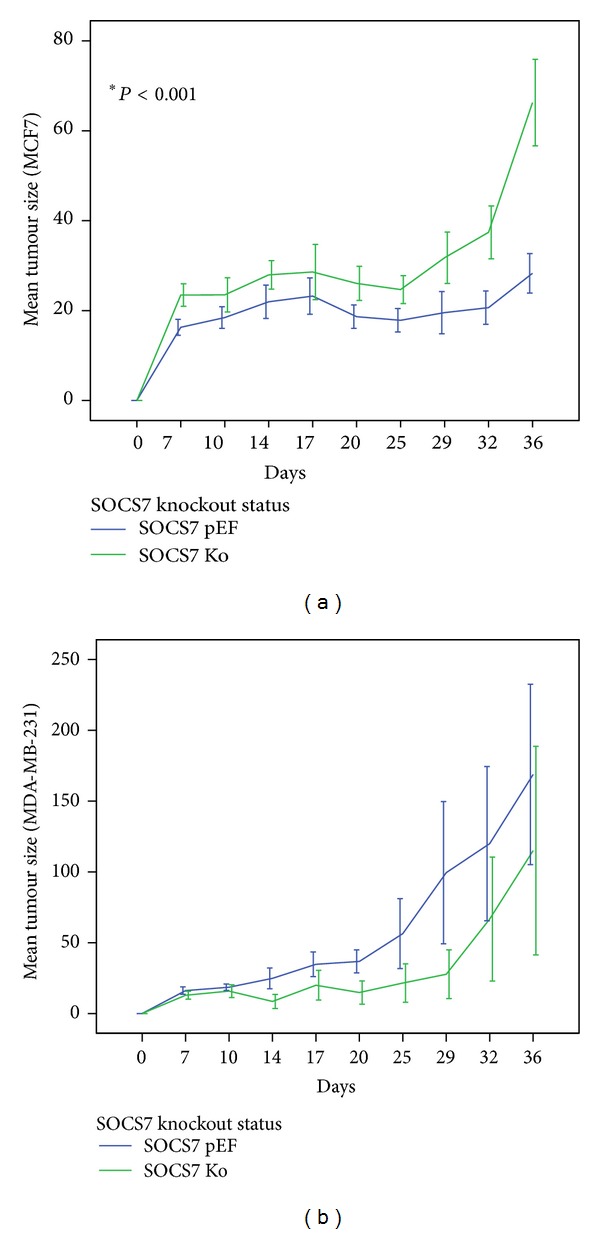
*In vivo* mammary tumour growth; (a) MCF7 tumours; (b) MDA-MB-231 tumours.

**Table 1:** Primers designed for amplifying the coding sequence of SOCS7.

Gene	Name of primer	Sequence of primers	Optimal annealing temperature
SOCS7 Set 1	SOCS7 ExF1	5′-ATGGTGTTCCGCAACGTG	55°C
	SOCS7 R8	5′-ACCAGGAAAGAACCATCTG	
SOCS7 Set 2	SOCS7 F2	5′-CCGAAAGTT CTACTACTATGAT	55°C
	SOCS7 R8	5′-ACCAGGAAAGAACCATCTG	
SOCS7 Set 3	SOCS7 F8	5′-CTCAAAGTGCCTTTTCTCC	55°C
	SOCS7 R8	5′-ACCAGGAAAGAACCATCTG	
SOCS7 Set 4	SOCS7 F8	5′-CTCAAAGTGCCTTTTCTCC	55°C
	SOCS7 ExR1	5′-CTACGTGGAGGGTTCCACCTCTT	
SOCS7 Set 5	SOCS7 F8	5′-CTCAAAGTGCCTTTTCTCC	55°C
	SOCS7 ExR2	5′-CTACGTGGAGGGTTCCACCTCT	
SOCS7 Set 6	SOCS7 F8	5′-CTCAAAGTGCCTTTTCTCC	55°C
	SOCS7 ExR3	5′-CTACGTGGAGGGTTCCACCTC	
SOCS7 Set 7	SOCS7 F8	5′-CTCAAAGTGCCTTTTCTCC	56°C
	SOCS7 ExR4	5′-CTACGTGGAGGGTTCCACCT	
SOCS7 Set 8	SOCS7 F8	5′-CTCAAAGTGCCTTTTCTCC	56°C
	SOCS7 ExR5	5′-CTACGTGGAGGGTTCCACC	
SOCS7 Set 9	SOCS7 F8	5′-CTCAAAGTGCCTTTTCTCC	55°C
	SOCS7 ExR6	5′-CTACGTGGAGGGTTCCA	

**Table 2:** Primers for synthesis of the SOCS7 ribozymes 1, 2, 3.

Ribozyme	Name of primer	Sequence of primers
SOCS7 ribozyme-1	SOCS7 RIB1F	5′-CTGCAGGCGGCTGGGGCTGCGGAGGGGGCGGCTGAGGAGCTGATGAGTCCGTGAGGA
	SOCS7 RIB1R	5′-ACTAGTGGGGGTTGCTGCCCGTGTCCGTGTTTCGTCCTCACGGACT
SOCS7 ribozyme-2	SOCS7 RIB2F	5′-CTGCAGGGGGCGGCTGAGGAGCTGATGAGTCCGTGAGGA
	SOCS7 RIB2R	5′-ACTAGTCGGTGGGGGTTGCTGCCCGTGTTTCGTCCTCACGGACT
SOCS7 ribozyme-3	SOCS7 RIB3F	5′-CTGCAGGTGCTGTGGGGGTTGGCTGTGCAGGCTGATGAGTCCGTGAGGA
	SOCS7 RIB3R	5′-ACTAGTGCTCCCATCCGAGCAGCTGAATTTCGTCCTCACGGACT

**Table 3:** The *in vitro* growth of MCF7 cells as measured by spectrophotometric light absorbance at a wavelength of 540 nm. Values represent mean (SD).

Cell lines		MCF7^ΔSOCS7^			*P*
	Incubation medium	Serum free only			
MCF7^WT^	Serum free only	**0.86 (0.12) **vs. 0.54 (0.04)			0.037
MCF7^pEF6^		**0.86 (0.12) **vs. 0.58 (0.1)			0.009

		HGF			
MCF7^WT^	HGF	**1.3 (0.25) **vs. 0.9 (0.1)			0.005
MCF7^pEF6^		**1.3 (0.25) **vs. 0.86 (0.1)			0.002

		Pretreatment with U73122 then HGF			
MCF7^WT^	Pretreatment with U73122 then HGF	**0.85 (0.02) **vs. 0.74 (0.25)			0.4
MCF7^pEF6^		**0.85 (0.02) **vs. 0.71 (0.17)			0.26

Cell lines		MCF7^WT^	MCF7^pEF6^	MCF7^ΔSOCS7^	*P*

			HGF		
MCF7^WT^	Serum free only	**0.9 (0.1) **vs. 0.54 (0.04)	**0.86 (0.1) **vs. 0.54 (0.04)		0.007 & 0.015
MCF7^pEF6^		**0.9 (0.1) **vs. 0.58 (0.1)	**0.86 (0.1) **vs. 0.58 (0.1)		0.015 & 0.03
MCF7^ΔSOCS7^				**1.3 (0.25) **vs. 0.86 (0.12)	0.002

**Table 4:** The *in vitro* growth of MDA-MB-231 cells as measured by spectrophotometric light absorbance at a wavelength of 540 nm. Values represent mean (SD).

Cell lines		MDA^ΔSOCS7^			*P*
	Incubation medium	HGF			
MDA^WT^	HGF	**1.8 (0.04)** vs. 1.3 (0.1)			<0.001
MDA^pEF6^		**1.8 (0.04)** vs. 1.4 (0.02)			<0.001

		Pretreatment with U73122 then HGF			
MDA^WT^	Pretreatment with U73122 then HGF	**1.3 (0.07)** vs. 1.2 (0.08)			0.49
MDA^pEF6^		**1.3 (0.07)** vs. 1.2 (0.14)			0.73

Cell lines		MDA^WT^	MDA^pEF6^	MDA^ΔSOCS7^	*P*

			HGF		
MDA^WT^	Serum free only	**1.3 (0.1)** vs. 0.9 (0.1)	**1.4 (0.02)** vs. 0.9 (0.1)		<0.001 (both)
MDA^pEF6^		**1.3 (0.1)** vs. 0.98 (0.05)	**1.4 (0.02)** vs. 0.98 (0.05)		<0.001 (both)
MDA^ΔSOCS7^				**1.8 (0.04)** vs. 1.3 (0.1)	<0.001

**Table 5:** The *in vitro* migration of MCF7 as measured by relative wound area (%). Values represent Mean (SD).

Cell lines		MCF7^ΔSOCS7^		*P*
	Incubation medium	HGF		
MCF7^pEF6^	HGF	**31.9 (19.6)** vs. 54.7 (15.5)		0.019

Cell lines		MCF7^pEF6^	MCF7^ΔSOCS7^	*P*

		HGF		
MCF7^pEF6^	Serum free only	**54.7 (15.5)** vs. 73.4 (9.8)		0.028
MCF7^ΔSOCS7^			**31.9 (19.6)** vs. 50.6 (5.9)	0.045

		Pretreatment with U73122 then HGF		
MCF7^pEF6^	HGF	**55.9 (17.2)** vs. 54.7 (15.5)		0.9
MCF7^ΔSOCS7^			**59.8 (20.3)** vs. 31.9 (19.6)	0.005

**Table 6:** The *in vitro* migration of MDA-MB-231 as measured by relative wound area (%). Values represent mean (SD).

Cell lines		MDA^ΔSOCS7^		*P*
	Incubation medium	HGF		
MDA^pEF6^	HGF	**34.9 (3.8)** vs. 58.5 (2)		0.01

Cell lines		MDA^pEF6^	MDA^ΔSOCS7^	*P*

		HGF		
MDA^pEF6^	Serum free only	**58.5 (2)** vs. 80.2 (4.5)		0.017
MDA^ΔSOCS7^			**34.9 (3.8)** vs. 54.3 (8)	0.017

		Pretreatment with U73122 then HGF		
MDA^pEF6^	HGF	**71.8 (4)** vs. 58.5 (2)		0.87
MDA^ΔSOCS7^			**61.2 (16.2)** vs. 34.9 (3.8)	0.007

**Table 7:** The *in vitro* migration rate of MCF7 (*μ*m/hr). Values represent Mean (SD).

Cell lines		MCF7^ΔSOCS7^		*P*
	Incubation medium	HGF		
MCF7^pEF6^	HGF	**97 (18)** vs. 62.9 (19.3)		0.008

Cell lines		MCF7^pEF6^	MCF7^ΔSOCS7^	P

		HGF		
MCF7^pEF6^	Serum free only	**62.9 (19.3)** vs. 37 (17.6)		0.026
MCF7^ΔSOCS7^			**97 (18)** vs. 83.9 (37.5)	0.252

		Pretreatment with U73122 then HGF		
MCF7^pEF6^	HGF	**46.9 (14.2)** vs. 62.9 (19.3)		0.184
MCF7^ΔSOCS7^			**60.3 (21.6)** vs. 97 (18)	0.005

**Table 8:** The *in vitro* migration rate of MDA-MB-231 (*μ*m/hr). Values represent Mean (SD).

Cell lines		MDA^ΔSOCS7^		*P*
	Incubation medium	HGF		
MDA^pEF6^	HGF	**222 (48.1)** vs. 104.2 (36.1)		<0.001

Cell lines		MDA^pEF6^	MDA^ΔSOCS7^	*P*

		HGF		
MDA^pEF6^	Serum free only	**104.2 (36.1)** vs. 37.1 (2.6)		0.008
MDA^ΔSOCS7^			**222 (48.1)** vs. 138.9 (13.9)	<0.001

		Pretreatment with U73122 then HGF		
MDA^pEF6^	HGF	**54.2 (10.2)** vs. 104.2 (36.1)		0.046
MDA^ΔSOCS7^			**59.3 (58.8)** vs. 222 (48.1)	<0.001

**Table 9:** MCF7 *in vivo* growth analysis (two-way ANOVA). Dependent variable: tumour size (MCF7).

Source	Type III sum of squares	Df	Mean square	*F*	Sig.	Partial eta squared
Corrected model	27431.367^a^	19	1443.756	10.008	.000	.576
Intercept	90065.079	1	90065.079	624.337	.000	.817
Phenotype	4387.925	1	4387.925	30.417	.000	.178
Days	18976.157	9	2108.462	14.616	.000	.484
Phenotype ∗ days	4067.285	9	451.921	3.133	.002	.168
Error	20196.016	140	144.257			

Total	137692.463	160				
Corrected total	47627.384	159				

^a^ *R* squared = .576 (adjusted *R* squared = .518).

**Table 10:** MDA-MB-231 *in vivo* growth analysis (two-way ANOVA). Dependent variable: tumour size (MDA-MB-231).

Source	Type III sum of squares	Df	Mean square	*F*	Sig.	Partial eta squared
Corrected model	296442.407^a^	19	15602.232	2.219	.005	.257
Intercept	268539.086	1	268539.086	38.200	.000	.238
Phenotype	25879.501	1	25879.501	3.681	.057	.029
Days	246104.941	9	27344.993	3.890	.000	.223
Phenotype ∗ days	20397.217	9	2266.357	.322	.966	.023
Error	857630.677	122	7029.760			

Total	1444538.200	142				
Corrected total	1154073.084	141				

^a^ *R* squared = .257 (adjusted *R* squared = .141).
